# Evaluating service user & carer experience of videoconferencing software during COVID-19 pandemic

**DOI:** 10.1192/bjo.2021.152

**Published:** 2021-06-18

**Authors:** Joseph MacDonnell

**Affiliations:** Kendray Hospital

## Abstract

**Aims:**

To evaluate service user and carer experience of use of videoconferencing software (Microsoft Teams) during MDT meetings.

To identify specific areas for improvement

To make changes based on these recommendations

**Method:**

2 surveys were distributed to inpatients and their carers on a functional Older Adults inpatient ward (n = 21), including quantitative and qualitiative questions. The results from these were compiled, and on review, mutliple recommendations for improvement were made.

**Result:**

90% of service users find it helpful to have family present over videoconferencing software during their MDT meetings, and 91% of carers feel involved and able to contribute when they do join in this way

81% of carers have the technology available at home to use such software, but only 55% of them feel confident using it. 73% need more information on its use.

60% of carers referenced poor staff skills with software as a barrier to its use, and 60% referenced poor organisation of meetings

2 service users raised issue with the size of a small laptop screen not allowing them to see who was actually present over MS Teams, although none were concerned with issues around confidentiality and the use of such software

Several service users, carers and members of community teams identified poor sound quality as an issue, both when joining over the software, and when present in the room.

**Conclusion:**

Widespread use of videoconferencing software such as MS Teams is likely to continue beyond the end of the COVID-19 pandemic. Through discussion with the ward team, the IT department, the training department, and the local council, multiple changes were made to the service, as below. These form a recommended list of areas for improvement in other services.

Availability of videoconferencing equipment (in addition to laptop)

Dedicated videoconferencing microphone/speaker to improve sound quality

Display screen

Webcam

Organisation of meetings

Designating a chairperson to admit and introduce all participants

Designating a meeting organiser to invite all necessary participants

Staff skills

Local audit of staff familiarity with software

Introduction of mandatory training for staff on use of software

Carer skills & access to equipment

Information and support available from well-trained staff

Liaison with other organisations including council and third sector about availablity of equipment loans and training for carers

